# *Fusarium falciforme* keratitis in a corneal transplant recipient treated successfully with fosmanogepix and intracameral amphotericin B

**DOI:** 10.1128/asmcr.00033-26

**Published:** 2026-08-11

**Authors:** Ashley Zeoli, Christine Shieh, Karen C. Bloch, Lili Tao, Alex Mainor, Christo Cimino, Breann Zeches, Augusto Dulanto Chiang

**Affiliations:** 1 Division of Infectious Diseases, Department of Medicine, Vanderbilt University Medical Center 12328, Nashville, Tennessee, USA; 2 Department of Ophthalmology, Vanderbilt University Medical Center 12328, Nashville, Tennessee, USA; 3 Department of Pathology, Microbiology, and Immunology, Vanderbilt University Medical Center 204907, Nashville, Tennessee, USA; 4 Vanderbilt Institute for Clinical and Translational Research, Vanderbilt University 5718, Nashville, Tennessee, USA; 5 Department of Pharmaceutical Services, Vanderbilt University Medical Center 12328, Nashville, Tennessee, USA; Pattern Bioscience, Austin, Texas, USA

**Keywords:** fusarium keratitis, fungal keratitis, fosmanogepix, antifungal, case report

## Abstract

**Background:**

Ocular fungal infections are a major cause of vision loss worldwide. *Fusarium* spp., a leading cause of fungal keratitis, is often difficult to treat. There are no current standardized treatment guidelines for fungal keratitis. We present a case of *Fusarium* spp. keratitis that was treated with fosmanogepix and intracameral amphotericin B, highlighting additional treatment options in difficult-to-treat cases. To our knowledge, this is the first report of fosmanogepix use in *Fusarium* keratitis.

**Case Summary:**

A 72-year-old man presented with 2 weeks of right eye pain and redness and was diagnosed with *Fusarium falciforme* keratitis via ocular fungal culture. He failed multiple topical, oral, and intraocular antifungal agents, and required corneal transplantation. Histopathology from his explant showed hyaline hyphae with occasional sickle-shaped conidia, consistent with *Fusarium* spp. The infection recurred in the corneal allograft despite intravitreal voriconazole, intraocular amphotericin B, and topical natamycin. Ultimately, he underwent successful treatment with fosmanogepix, a first-in-class antifungal agent.

**Conclusion:**

Our case illustrates the challenges of managing *Fusarium* keratitis and the potential role of novel antifungal agents, such as fosmanogepix, as adjuncts to established intraocular and topical therapy in the setting of clinical failure.

## INTRODUCTION

Fungal keratitis is a medical emergency and a common cause of monocular blindness worldwide (1). The disease is seen mostly in tropical and subtropical climates and is associated with agricultural eye trauma (2). Other risk factors include contact-lens use, topical steroid use, and ocular surgery (3). The most common etiologies are *Aspergillus* spp. *and Fusarium* spp., with *Fusarium* spp. being the leading cause of filamentous fungal keratitis (4). The initial infection may be asymptomatic followed by photophobia, pain, and discharge days to weeks later (4). Diagnosis involves culture and sometimes biopsy. Treatment includes antifungal agents and corneal transplant if perforation occurs (4).

Fosmanogepix (prodrug, active moiety manogepix) is the first member of the “gepix” class of antifungals and is in Phase 3 clinical development for invasive mold infections (NCT06925321) and invasive candidiasis (NCT05421858). Fosmanogepix has *in vitro* and *in vivo* activity against a broad spectrum of yeasts (except for *Candida krusei*) and molds, including *Fusarium* spp. (5–7). Although not currently Food and Drug Administration (FDA) approved, fosmanogepix is available via named-patient expanded access by Basilea Pharmaceutica International Ltd. There are no reported cases to date of its use in *Fusarium* keratitis.

## CASE PRESENTATION

A previously healthy 72-year-old male presented with 2 weeks of right eye pain and redness. He sustained superficial ocular trauma from a cornstalk while farming 3 weeks prior and subsequently slept in his contact lenses. He delayed care until worsening symptoms, at which time a large corneal ulcer was noted. Initial bacterial and fungal ocular cultures were negative. He was prescribed topical vancomycin, ceftazidime, and prednisone eyedrops. A detailed clinical course can be found in Table 1.

**TABLE 1:** Clinical course, microbiology, and treatment timeline^*a*^

Day	Clinical finding	Antimicrobial agents	Culture results
Day 0	Diagnosed with corneal ulcer	Topical ceftazidime, vancomycin	
Day 5	Ulcer worsening	Topical natamycin, ceftazidime, vancomycin	
Day 6	Corneal scraping culture obtained	Topical natamycin, vancomycin, tobramycin	Corneal scraping: growth of *Fusarium* spp. after 3 days
Day 24	Diagnosed with perforated ulcer	Topical voriconazole, natamycin, moxifloxacin	
Day 27	Penetrating keratoplasty	Topical voriconazole, natamycin, moxifloxacin	Corneal tissue: finalized negative
Day 38	New opacity		
Day 41	Diagnosed with vitritis/endophthalmitis	Intravitreal injection with vancomycin, ceftazidime, voriconazole	
Day 45	Exam felt to be improved, continued clinical monitoring		
Day 48	Worsening symptoms (edema, pain) and exam with increased debris	Intravitreal voriconazole	
Day 49	Infectious diseases consulted	Voriconazole 300 mg PO BID started	
Day 53		Intracameral amphotericin B	Intracameral aspirate culture (+) *Fusarium* spp. after 4 days
Day 60		Intracameral amphotericin B	
Day 67		Intracameral amphotericin B	
Day 81	Exam with intraocular fibrotic membrane covering inferior half of iris and swelling of corneal transplant		
Day 87		Fosmanogepix started, voriconazole stopped	
Day 105		Patient self-decreased fosmanogepix dose by half	
Day 115	No evidence of recurrent infection on examination	Fosmanogepix discontinued	

^*a*^ Patient’s clinical timeline with associated examination findings and antimicrobial regimen received, as well as culture results (listed at the collection date) throughout his treatment course. Please see main text for further details. Day 0 represents the day of initial presentation.

**Fig 1:**
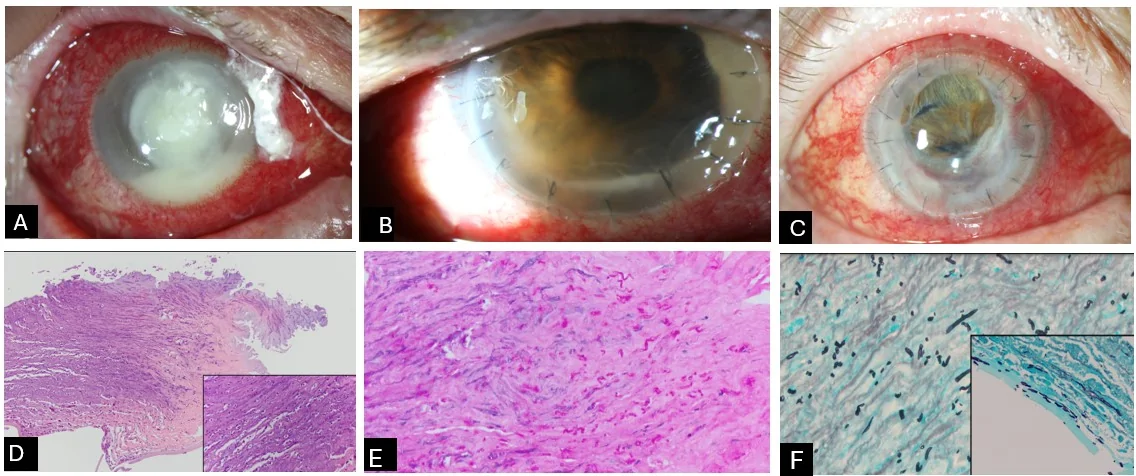
Clinical and histopathology images. (**A**) Initial presentation with large corneal ulcer and hypopyon. (**B**) Early infiltrate after cornea transplantation. (**C**) Cornea transplant after clearance of infection, with residual fibrotic membrane and inflammation. (**D**) (H&E, original magnification 100×). Corneal tissue with epithelial absence, abnormal stroma, intact Descemet’s membrane, and lack of endothelial cells. The stroma is largely necrotic and diffusely infiltrated by neutrophils and macrophages (inset, original magnification 400×). (**E and F**) (**E**) Periodic acid-Schiff (PAS) (original magnification 400×) and (**F**) Grocott methenamine silver (GMS) (**B**, original magnification 600×) highlight numerous septate fungal hyphal forms with acute-angle branching. Focal extension through Descemet’s membrane is identified (inset, original magnification 200×).

Over the following 2 weeks, his vision declined to light perception, with progressive ulceration. Prednisone eyedrops were discontinued, topical natamycin 5% was initiated, and he was referred to our facility. Examination revealed an 8 × 7 mm central corneal ulcer with a large hypopyon (Fig. 1A). Corneal scraping was performed and sent for bacterial and fungal culture. The patient initially opted out of confocal microscopy. Natamycin was continued, and topical tobramycin 0.3% with oral prednisone (40 mg daily) was added.

**Fig 2:**
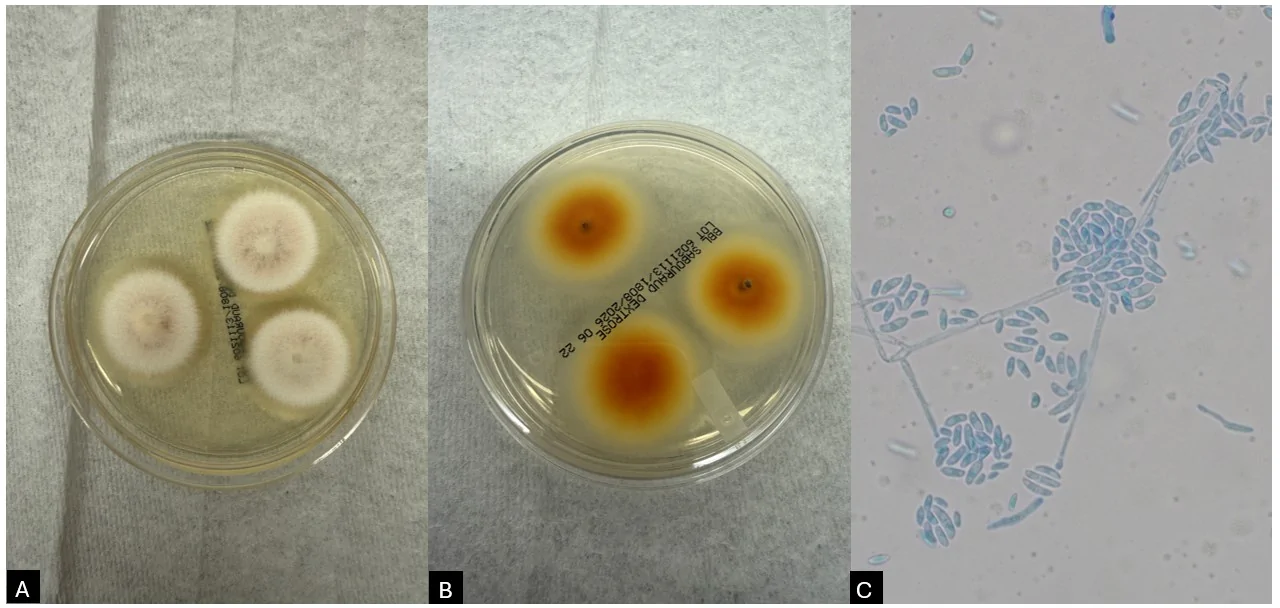
Macroscopic and microscopic features of the *Fusarium falciforme* isolate. (**A**) Front view of the colonies grown on Sabouraud dextrose agar (SAB) plate, displaying floccose, pale-yellow colonies with a light-colored margin. (**B**) Reverse view of the colonies, showing the production of a yellow-orange pigment. (**C**) Lactophenol blue staining of the isolate revealing septate hyaline hyphae and erect conidiophores bearing clusters of sickle-shaped conidia.

Gram stain revealed a small number of nucleated cells with no microorganisms; KOH staining was not performed. The bacterial culture of the corneal scraping showed no growth. However, after 3 days of incubation at 30°C, a mold with pale yellow color grew on the Sabouraud dextrose agar (SAB) (Fig. 2A and B). Lactophenol blue staining of the mold revealed septate hyaline hyphae and sickle-shaped conidia, consistent with *Fusarium* spp. (Fig. 2C). Species-level identification was achieved by DNA sequencing of RNA polymerase II (*RPB2*) and translation elongation factor (*TEF*) at the UT Health San Antonio Fungus Testing Laboratory, which identified the organism as *Fusarium falciforme*. Despite initial symptomatic improvement, he developed a corneal perforation 2 weeks later. His local ophthalmologist performed emergent corneal gluing until he underwent therapeutic penetrating keratoplasty (PK).

Histopathology of the corneal tissue demonstrated necrotizing fungal keratitis that extended focally through Descemet’s membrane. Fungal special staining using periodic acid-Schiff (PAS) and Gomori methenamine silver (GMS) highlighted septate, non-pigmented hyphae with acute-angle branching, along with occasional canoe-shaped conidia (Fig. 1D, E and F). Fungal and bacterial culture of corneal tissue obtained from PK showed no growth. After PK, he was treated with topical natamycin, 1% voriconazole, and 0.5% moxifloxacin eye drops, along with oral prednisone taper. Given the concern for residual organisms, topical tacrolimus 0.03% was added off-label to preserve the graft while limiting steroid exposure.

By post-operative week 2, he developed a new corneal opacity with increased photophobia, pain, and tearing (Fig. 1B). Examination revealed vitritis. He underwent an intravitreal tap and injection of vancomycin 1 mg, ceftazidime 2 mg, and voriconazole 0.1 mg. Vitreous cultures remained negative for *Fusarium*. By post-operative week 3, with worsening pain and possible satellite lesions, he received repeat intravitreal and intracameral voriconazole. Fungal culture from intracameral aspirate fluid again grew *F. falciforme*, while vitreous cultures remained negative.

Infectious Diseases was consulted for systemic antifungals, and oral voriconazole (300 mg BID) was started post-operative week 3 along with intracameral amphotericin B 5 mcg injection post-operative week 4. He continued weekly amphotericin B injections for three doses and systemic voriconazole for 5 weeks. Despite this, he continued to have concerning signs of residual infection in his cornea graft. He was therefore transitioned to fosmanogepix (1,000 mg IV BID day 1, followed by 600 mg IV daily days 2 to 3, then 800 mg PO daily). Antifungal susceptibility testing (AST) was performed at a reference laboratory using broth microdilution, which showed minimum inhibitory concentrations (MICs) of >16 mg/mL for both voriconazole and posaconazole and a minimum effective concentration (MEC) of <0.008 µg/mL for fosmanogepix. Susceptibility for amphotericin B was not obtained.

After initiation of fosmanogepix and three weekly intracameral amphotericin B treatments, his pain and photosensitivity resolved. He completed 29 days of fosmanogepix, although he decreased his dosing by half during the last week due to flank discomfort. Subsequent evaluation revealed a benign liver cyst which was thought to be the cause of his pain. Dual topical natamycin and topical voriconazole were maintained through the perioperative and post-operative periods, initially every 2 h while awake, then gradually tapered to four times daily over the ensuing months based on clinical improvement. Voriconazole eyedrops were discontinued 3 months after presentation. Natamycin was continued for another 2.5 months and then discontinued after the absence of fungal elements was confirmed by confocal microscopy.

After resolution of his infection, his corneal transplant remained clear, albeit with residual fibrotic membrane and inflammation (Fig. 1C). At follow-up 2 years later, there was no recurrence of fungal keratitis. However, his initial corneal graft failed following glaucoma tube and cataract surgery, and additional surgery is planned.

## DISCUSSION

This case highlights the multifactorial approach required for refractory *Fusarium* keratitis, particularly in the setting of clinical failure after corneal transplant, and provides real-world context for combined use of topical, intraocular, and systemic antifungal therapy.

To our knowledge, there are no reported cases of *Fusarium* keratitis treated with fosmanogepix. In this patient, *Fusarium* infection was likely related to ocular inoculation with plant material, with continued contact lens wear contributing to the delayed presentation. As in other reports (8), systemic antifungal therapy was pursued given progressive disease despite topical and intraocular agents. *Fusarium falciforme* is a member of the *Fusarium solani* complex and, in addition to fungal keratitis, can cause disseminated invasive fusariosis, onychomycosis, and skin and soft tissue infections (9). Although *Fusarium* spp., *Aspergillus* spp., and *Candida* spp. account for most fungal keratitis cases, other culprit organisms include dematiaceous molds and *Purpureocillium lilacinum*, an emerging pathogen causing severe ocular infections seen in contact lens wearers and immunosuppressed individuals (10, 11).

First-line treatment of fungal keratitis typically involves topical antifungal therapy with natamycin or voriconazole (4). Natamycin remains the only FDA-approved topical antifungal agent for ocular fungal infections, although other agents such as voriconazole and amphotericin B are also used. Its large molecular weight limits penetration into deeper ocular tissues, often necessitating prolonged treatment courses (12).

Prior to the Mycotic Ulcer Treatment Trial I (MUTT I), few adequately powered studies evaluating treatment options for fungal keratitis existed. MUTT I, a randomized controlled trial including 128 cases of *Fusarium* keratitis, demonstrated that natamycin was associated with better visual outcomes and lower risk of corneal perforation compared to topical voriconazole (13). A 2015 Cochrane review of fungal keratitis synthesized data from multiple trials and reached a similar conclusion (3). Oral antifungal therapy with voriconazole and posaconazole was studied in severe or refractory fungal keratitis (14). MUTT II evaluated oral voriconazole as an adjunctive therapy to topical therapy and found no overall benefit in patients. A subgroup analysis by species, however, suggested a possible (although not statistically significant) advantage in *Fusarium* cases (15).

In refractory *Fusarium* cases, intrastromal and intracameral injections can achieve higher local drug concentrations. While evidence remains limited, intracamerally administered amphotericin B can aid in the treatment of *Fusarium* keratitis with hypopyon (16). Unlike bacterial keratitis, where hypopyon typically represents sterile inflammation, the presence of a hypopyon in fungal keratitis is clinically significant. Fungal organisms can penetrate an intact Descemet’s membrane in the cornea, and the presence of hypopyon suggests intraocular invasion. In this patient, intracameral amphotericin B was administered. Given the timing of clinical improvement, we believe that this might have contributed to his recovery.

The optimal treatment for *Fusarium* keratitis after topical therapy failure remains undefined. Sharma et al. have proposed a stepwise escalation beginning with topical natamycin, followed by topical voriconazole, intrastromal voriconazole, and finally oral voriconazole in the absence of improvement (17). Our patient’s course mirrored this approach, escalating through topical, intraocular, systemic, and ultimately novel antifungal therapy after PK for cornea perforation.

Management was further complicated by clinical failure; thus, the decision was made to transition systemic antifungal therapy from voriconazole to fosmanogepix. As adjunct information, the *Fusarium falciforme* isolate MICs for voriconazole and posaconazole were greater than the highest concentration tested (>16 mg/mL) but displayed low MEC to fosmanogepix (MEC ≤0.008 µg/mL). The interpretation of antifungal susceptibility remains challenging in many mold infections, including *Fusarium* keratitis. Breakpoints are not available for most molds, and *in vitro* AST often does not correlate with clinical response, as clinical success can be seen despite elevated MICs. Notably, *Fusarium* spp. often shows elevated MECs to most common antifungal drugs (triazoles, amphotericin B, and echinocandins). In select cases, AST can aid in decision-making when there is concern for clinical failure or support the need to transition to a different antifungal agent.

Fosmanogepix is a first-in-class novel oral antifungal. Its prodrug is metabolized to manogepix, which inhibits the fungal Gwt1 protein, critical for anchoring mannoproteins to the fungal cell membrane and wall (18). This agent was selected for its favorable tissue distribution, including ocular penetration, and reported efficacy in disseminated *Fusarium* infections (5, 19, 20).

Fosmanogepix was discontinued after 29 days, once there was no clinical evidence of infection and after a treatment duration similar to that used in MUTT II *Fusarium* keratitis (20 days of systemic voriconazole) (15). Although the patient’s improvement coincided with fosmanogepix therapy, the contribution of intracameral amphotericin B must also be acknowledged.

In conclusion, this is the first report of fosmanogepix use in refractory *Fusarium* keratitis after corneal transplant. Our case illustrates the challenges of managing fungal keratitis cases refractory to topical and systemic therapy and the potential role of novel antifungal agents as adjuncts to established intraocular and topical therapy.

## Data Availability

The data underlying this article will be shared on reasonable request to the corresponding author.
